# *xcore*: an R package for inference of gene expression regulators

**DOI:** 10.1186/s12859-022-05084-0

**Published:** 2023-01-11

**Authors:** Maciej Migdał, Takahiro Arakawa, Satoshi Takizawa, Masaaki Furuno, Harukazu Suzuki, Erik Arner, Cecilia Lanny Winata, Bogumił Kaczkowski

**Affiliations:** 1 Laboratory of Zebrafish Developmental Genomics, International Institute of Molecular and Cell Biology in Warsaw, Warsaw, Poland; 2 RIKEN Center for Integrative Medical Sciences, Yokohama, 230-0045 Japan; 3 Present Address: GSK, Gunnels Wood Rd, Stevenage, SG1 2NY UK; 4 Present Address: Data Sciences and Quantitative Biology, Discovery Sciences, AstraZeneca R&D, Cambridge, UK

**Keywords:** Gene expression, Gene regulation, Regression, Transcription factors, ChIP-seq

## Abstract

**Background:**

Elucidating the Transcription Factors (TFs) that drive the gene expression changes in a given experiment is a common question asked by researchers. The existing methods rely on the predicted Transcription Factor Binding Site (TFBS) to model the changes in the motif activity. Such methods only work for TFs that have a motif and assume the TF binding profile is the same in all cell types.

**Results:**

Given the wealth of the ChIP-seq data available for a wide range of the TFs in various cell types, we propose that gene expression modeling can be done using ChIP-seq “signatures” directly, effectively skipping the motif finding and TFBS prediction steps. We present *xcore*, an R package that allows TF activity modeling based on ChIP-seq signatures and the user's gene expression data. We also provide *xcoredata* a companion data package that provides a collection of preprocessed ChIP-seq signatures. We demonstrate that *xcore* leads to biologically relevant predictions using transforming growth factor beta induced epithelial-mesenchymal transition time-courses, rinderpest infection time-courses, and embryonic stem cells differentiated to cardiomyocytes time-course profiled with Cap Analysis Gene Expression.

**Conclusions:**

*xcore* provides a simple analytical framework for gene expression modeling using linear models that can be easily incorporated into differential expression analysis pipelines. Taking advantage of public ChIP-seq databases, *xcore* can identify meaningful molecular signatures and relevant ChIP-seq experiments.

**Supplementary Information:**

The online version contains supplementary material available at 10.1186/s12859-022-05084-0.

## Background

Gene expression profiling is often performed to elucidate the transcriptional regulators in a given system/perturbation. A common approach is to use transcription factor motifs to computationally predict the TFBS within promoter regions of known genes. The “motif activity” is then inferred based on gene expression profiles [1–3]. Although such methods are quite simplistic, they proved useful for the identification of key molecular regulators [1, 2, 4, 5]. The limitations are that many TFs do not have a defined motif and some binding events may be specific to a particular biological context.

ReMap [6] and ChIP-Atlas [7] provide a wealth of uniformly processed ChIP-seq data (genome-wide peaks) for TFs but also other transcriptional regulators including transcriptional coactivators and chromatin-remodeling factors. Currently, only a limited number of tools exist that tap into these databases. Two examples are Lisa [8] identifying the most likely transcriptional regulators in an experiment based on user-supplied gene expression information, and Virtual ChIP-seq program [9] that can predict the binding of individual TF in a cell type of interest based on gene expression information. However, to our knowledge, there are no published methods that take advantage of this data to directly model the activity of transcriptional regulators.

**Fig. 1:**
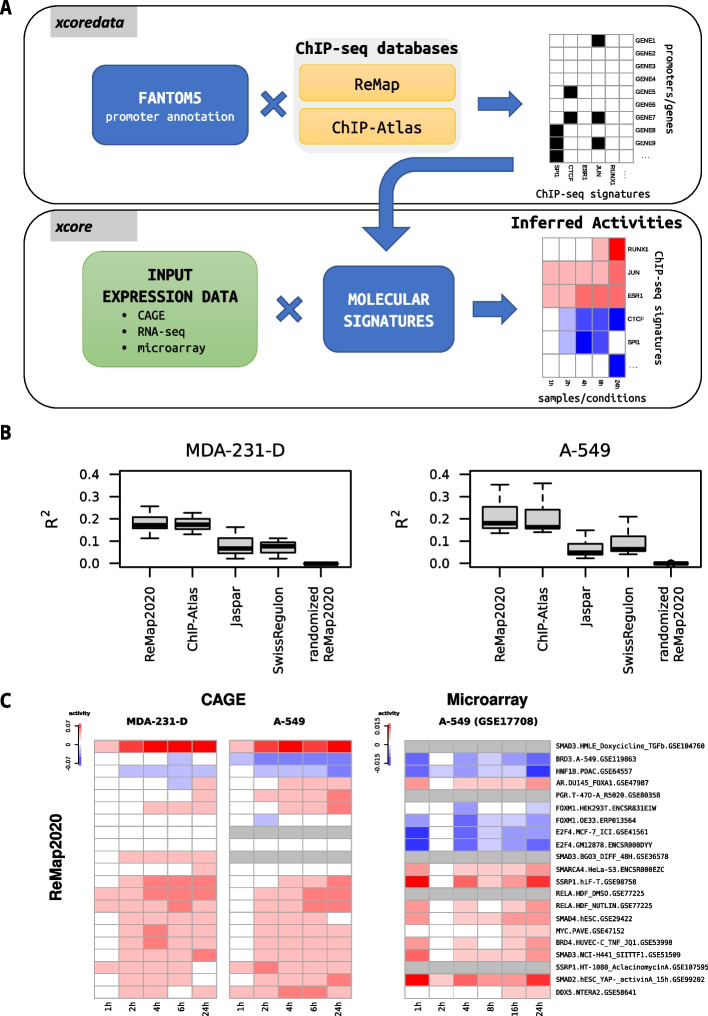
Inferring transcription factors activities from gene expression during TGFβ induced EMT in A-549 and MDA-231-D cell lines. **A** Flowchart depicting *xcore* and *xcoredata* functionalities. **B** Boxplots showing R^2^ values for gene expression prediction models constructed using different molecular signature sets: Motif-based (Jaspar, SwissRegulon) and ChIP-seq based (ReMap2020, ChIP-Atlas). Each boxplot shows R^2^ values pooled across all the replicates. Models were trained and evaluated in tenfold cross-validation on individual replicates, using data on gene expression changes between 0 and 24 h after treatment in our newly generated TGFβ induced EMT experiment performed in A-549 and MDA-231-D cell lines. **C** Heatmap showing the dynamics of TF activities during TGFβ induced EMT. Heatmaps on the left present TF activities estimated using CAGE data from our newly generated TGFβ induced EMT experiment performed on A-549 and MDA-231-D cell lines. Heatmap on the right depicts TF activities estimated using previously published microarray data from the TGFβ induced EMT experiment performed on A-549 cell lines. The TF activities were calculated in the reference to 0 h time point. Only the top-scoring ReMap2020 signatures are shown. Grey color designates NA values

Here, we propose to use the publicly available ChIP-seq data to directly represent the genome-wide occupancy of regulators. We intersected the peaks with promoter regions and used linear ridge regression to infer the regulators associated with observed gene expression changes (Fig. 1A). The advantage of this approach is the direct integration of gene expression profiles with experimental TF binding data. We provide (a) processed and pre-computed, ChIP-seq based molecular signatures (*xcoredata*), and (b) methodology for activity modeling (*xcore*). The framework is implemented as an R package (available in Bioconductor) and integrates smoothly with commonly used differential expression workflows like edgeR [10] or DESeq2 [11].

## Implementation

### Expression data processing

*Xcore* takes promoter or gene expression counts matrix as input, the data is then filtered for lowly expressed features, normalized for the library size and transformed into counts per million (CPM) using edgeR [10]. Users need to designate the base-level samples by providing an experiment design matrix. These samples are used as a baseline expression when modeling changes in gene expression. *xcore* implements promoter- and gene-level analyses, using either promoter or gene expression data. In our experience we found promoter-level analysis to provide better results (Additional file 1: Fig. S1). Cap Analysis Gene Expression (CAGE) data is an input of choice for promoter level analysis. However, *xcore* can be used with other types of expression data such as microarray or RNA-seq data to perform gene-level analysis. Promoter-level analysis based on RNA-seq data is possible in principle but currently not implemented.

### Molecular signatures

A second input consists of molecular signatures describing known transcription factors’ binding preferences within the promoter's vicinity. We provide sets of precomputed molecular signatures with *xcoredata*, the accompanying data package. The signatures were obtained by downloading all ChIP-seq data from ReMap2020 [6] and ChIP-Atlas [7] and intersecting it against ± 500 nt window of know promoter regions, defined based on FANTOM5’s hg38 annotation [12]. The signatures can also be easily constructed using *xcore* by providing predicted TFBS or custom ChIP-seq peaks (see *xcore* user guide). Detailed information on the molecular signatures construction can be found in Extended Materials and Methods (Additional file 3).

### Expression modeling

In *xcore* we describe the relationship between the expression (Y) and molecular signatures (X) using linear model formulation:

$$Y = \mu + \beta_{0} + \beta_{1} X_{1} + \cdots + \beta_{p} X_{p}$$

where Y is a sample expression level, µ is the basal expression level, β_0_ is the intercept, β_j_ is a j-th molecular signature activity and X_j_ is a j-th molecular signature.

Here, we are interested in finding the unknown molecular signatures’ activities (β) that describe the effect of molecular signature (X) on expression (Y). By including µ in the above equation we effectively model the change in expression between the basal expression level and the corresponding sample. Models are trained using penalized linear regression. In particular, we use ridge regression [13] as it allows us to take advantage of an existing significance testing methodology [14]. We observed ridge regression to work equally well to lasso and elastic net regression (Additional file 2: Fig. S2C). In practice, to fit our linear models we use the popular R package glmnet [15]. For each sample, that is for each time point and replicate, a separate model is trained using sample change in expression and molecular signatures shared at the experiment scale. In layman’s terms, for each sample, we are seeking to find a combination of ChIP-seq based signatures that best explains the observed changes in gene expression. For each model, the ridge regression λ tuning parameter is found separately using the cross-validation technique (CV). By default tenfold CV is used, and λ value giving the smallest mean squared error is selected.

Next, the estimated molecular signatures’ activities can be tested for significance. In short, using matrix formulation the ridge regression estimator is defined as

$$\hat{\beta }^{\lambda } = \left( {X^{\prime } X + \lambda I} \right)^{ - 1} X^{\prime } Y$$

where $X$ is our molecular signatures matrix, $\lambda$ is a ridge regression tuning parameter, and $Y$ is a vector of our sample’s changes in expression. Then, the estimate of β^λ^ standard error is calculated from the following:

$$Var\left( {\beta^{\lambda } } \right) = \hat{\sigma }^{2} (X^{\prime } X + \lambda I)^{ - 1} X^{\prime } X(X^{\prime } X + \lambda I)^{ - 1} ,$$

$$\hat{\sigma }^{2} = \frac{{\left( {Y - X\hat{\beta }^{\lambda } } \right)^{\prime } \left( {Y - X\hat{\beta }^{\lambda } } \right)}}{\nu }$$

where ν is the residual effective degrees of freedom. The significance of the individual molecular signatures' activities can be then tested using a test of significance for ridge regression coefficients. For further details, we refer interested readers to [14].

To summarize the results from individual replicates, following the procedure described in [16], the obtained estimates and their standard errors are pooled across the replicates by calculating their weighted mean with variance-defined weights and weighted mean error:

$$\overline{x} = \frac{{\mathop \sum \nolimits_{i = 1}^{n} \frac{{x_{i} }}{{\sigma_{i}^{2} }}}}{{\mathop \sum \nolimits_{i = 1}^{n} \frac{1}{{\sigma_{i}^{2} }}}},\quad \sigma_{{\overline{x}}} = \sqrt {\frac{1}{{\mathop \sum \nolimits_{i = 1}^{n} \frac{1}{{\sigma_{i}^{2} }}}}}$$

Using this result, we calculate a Z-score for each molecular signature and time-point.

Finally, molecular signatures are ranked based on their overall Z-score across the time-points calculated using Stouffer’s Z method [17].

### Linear regression models comparison

To compare different models, coefficients of determination (R^2^) were calculated for models trained on individual replicates at selected time points using tenfold cross-validation and pooled across replicates. Additional information on this procedure is provided in Extended Materials and Methods (Additional file 3).

## Results

We used *xcore* to perform gene expression modeling analysis in the context of three CAGE datasets: (a) newly generated transforming growth factor beta (TGFβ) induced epithelial-mesenchymal transition (EMT) experiment performed in A-549 and MDA-231-D cell lines, (b) previously published FANTOM5’s rinderpest infection time-course dataset performed in 293SLAM and COBL-a cell lines using native and recombinant rinderpest virus lacking accessory V and C proteins [12], (c) previously published FANTOM5’s Human H3 embryonic stem cells differentiated to cardiomyocytes time-course dataset [12] and a microarray dataset: previously published TGFβ induced EMT in A-549 cell line (GSE17708) [18]. Detailed information on the procedures used to process the raw CAGE data can be found in Extended Materials and Methods (Additional file 3).

### ChIP-seq molecular signatures provides better model performance

We compared the models built using ChIP-seq signatures (ReMap2020 and ChIP-Atlas) vs motif-based signatures (Jaspar and SwissRegulon). The models based on ChIP-seq signatures showed on average higher R^2^ values, which reflects the proportion of variance explained by the model and overall “goodness of fit”. In particular, modeling expression between 0 and 24 h after TGFβ treatment in our novel MDA-231-D dataset yielded an average R^2^ of 0.179 for ChIP-seq signatures and 0.077 for motif signatures. For comparison the randomized version of ReMap2020 molecular signature yielded R^2^ close to 0 (Fig. 1B, Additional file 2: Fig. S2B).

### xcore recovers biologically relevant expression regulators

**Fig. 2:**
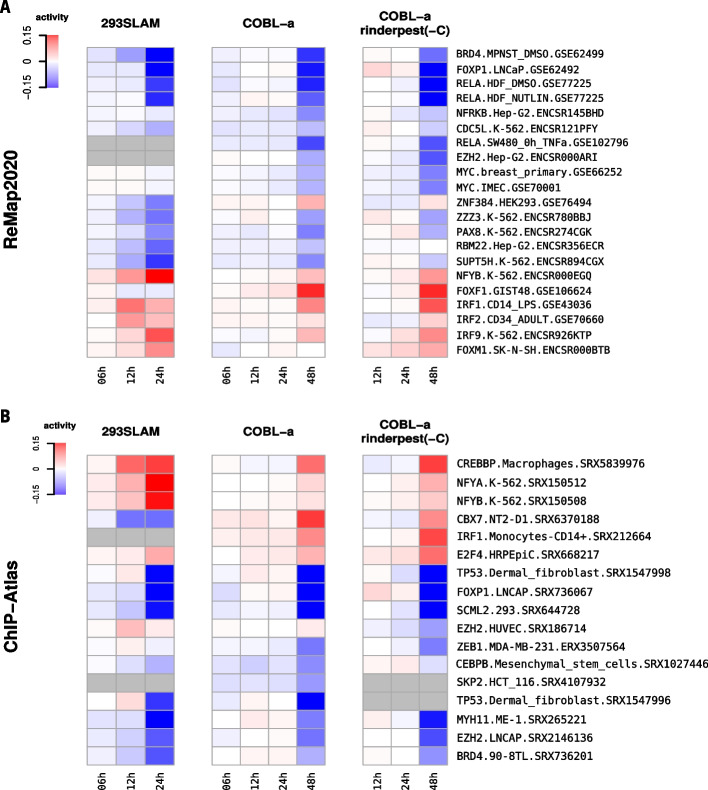
Estimating transcription factors activities from gene expression during rinderpest infection in 293SLAM and COBL-a cell lines. **A**, **B** Heatmaps presenting TF activities of the most significant molecular signatures inferred using FANTOM5’s rinderpest infection time-series dataset. The underlying experiments were performed in 293SLAM and COBL-a cell lines using native and recombinant rinderpest virus lacking accessory V and C proteins (rinderpest(-C)). Results obtained using ReMap2020 and ChIP-Atlas based molecular signatures are displayed on the top and bottom panels respectively

To investigate the biological relevance of the obtained results, we looked at the top-scoring signatures from ReMap2020 (Fig. 1C) and ChIP-Atlas (Additional file 2: Fig. S2A) in TGFβ induced EMT datasets. Among those, we identified known key TFs involved in the TGFβ pathway such as *SMAD2/3/4* [19], *SSRP1*, *HNF1B* [20], *DDX5* [21] or *RELA* [22]. Other well-known EMT-linked TFs also returned as significant including *ZEB1*, *SNAI2*, *TBX3*, *SOX4* (Additional file 4: Table S1, Additional file 5: Table S2, Additional file 6: Table S3). In case of FANTOM5’s rinderpest infection dataset, top-scoring ReMap2020 and ChIP-Atlas signatures (Fig. 2, Additional file 7: Table S4) showed several TFs involved in the closely related measles infection pathway, including *RELA*, *IRF9, TP53* (KEGG PATHWAY:map05162) [23]. For human H3 embryonic stem cells differentiated to cardiomyocytes time-course dataset, a number of known heart development regulators were found among top-scoring ReMap2020 and ChIP-Atlas signatures (Additional file 8: Table S5), such as *JARID2, SMAD3, NKX2-5* (GO:0007507) [24]*.*

### Comparison with the state-of-the-art tools

We compared our results with state-of-the-art motif-based gene expression prediction framework ISMARA [1] and Lisa program which predicts the most likely transcriptional regulators from gene expression data based on ChIP-seq and chromatin accessibility data available in Cistrome Data Browser [25]. While ISMARA is conceptually similar and was inspirational to *xcore*, Lisa takes a different approach. Using a user supplied list of differentially expressed genes, Lisa first selects a subset of relevant experiments describing chromatin state (H3K27ac ChIP-seq or DNase-seq) using lasso regression. Next it identifies the most relevant TF using *in-silico* deletion technique [8]. To compare with our results, we used both tools on our novel TGFβ induced EMT, rinderpest infection and embryonic stem cells differentiated to cardiomyocytes datasets. We have run ISMARA in RNA-seq mode with a genome version hg38 and no miRNA using raw FASTQ files for our novel TGFβ induced EMT dataset and BAM files available in FANTOM5 study [12] mapped against genome version hg38 for the other datasets. To use Lisa we performed differential expression analysis using edgeR [10] between the most extreme time points in our time-course datasets. Then lists of 100 most significant up- (logFC > 0) and 100 most significant down-regulated (logFC < 0) genes were submitted to Lisa. Next, we compared the results from all tools with a list of related transcriptional regulators. We constructed lists of related transcriptional regulators for each dataset using Gene Ontology term *regulation of epithelial to mesenchymal transition* (GO:0010717), KEGG pathway *Measles* (map05162) and Gene Ontology term *heart development* (GO:0007507) by including only regulators available in the references of all tools. The number of EMT related transcriptional regulators recovered among the top-scoring signatures was higher for *xcore* and Lisa than ISMARA (Table 1). In case of rinderpest infection (Table 2) Lisa recovered the highest number of related TF in 293SLAM cell line. In the case of COBL-a and COBL-a rinderpest(-C) analyzes *xcore* found one more TF than ISMARA and Lisa. Finally, for embryonic stem cells differentiated to cardiomyocytes (Table 3) Lisa was able to find the highest number of related TF, while *xcore* and ISMARA found the same number of related TF.

**Table 1:** Recovering epithelial to mesenchymal transition transcriptional regulators

Top signatures	A-549				MDA-231-D			
	ISMARA	Lisa	*xcore*		ISMARA	Lisa	*xcore*	
			ReMap2020	ChIP-Atlas			ReMap2020	ChIP-Atlas
1–10	SMAD4	SMAD3, SMAD4, GATA3	SMAD3, SMAD2	SMAD3, SMAD2, SMAD4		SMAD4, SMAD3	SMAD3, SMAD4	SMAD3, SMAD4, SMAD2, GATA3
11–50		FOXA2, FOXA1	EZH2, GATA3, SMAD4	EZH2, FOXA2	SMAD4	GATA3	SMAD2	EZH2, FOXA2, FOXA1
51–100	GATA3, FOXA1	NKX2-1, TCF7L2	FOXA1, FOXA2, NKX2-1	FOXA1, GATA3		FOXA1, NKX2-1	EZH2, FOXA1	

Table summarizing EMT-related transcriptional regulators recovered by ISMARA, Lisa and *xcore* among their top-scoring signatures based on TGFβ induced EMT CAGE datasets. The list of EMT-related transcriptional regulators used to assess the recovery was constructed using Gene Ontology term *regulation of epithelial to mesenchymal transition* (GO:0010717) by including only regulators available in the references of all tools

**Table 2:** Recovering measles infection transcriptional regulators

Top signatures	293SLAM				COBL-a				COBL-a rinderpest(-C)			
	ISMARA	Lisa	*xcore*		ISMARA	Lisa	*xcore*		ISMARA	Lisa	*xcore*	
			ReMap2020	ChIP-Atlas			ReMap2020	ChIP-Atlas			ReMap2020	ChIP-Atlas
1–10	STAT2, IRF3, IRF9	TP53, STAT1	IRF9	TP53			RELA	TP53	STAT2		RELA, IRF9	TP53
11–50	RELA, JUN	STAT3, IRF9, RELA, STAT5B, STAT2	RELA, STAT1	NFKB1, RELA, STAT2	FOS	TP53, STAT3	TP53, FOS	STAT1, RELA, JUN	IRF3	TP53, STAT3	STAT5A	NFKB1, JUN
51–100	NFKB1, FOS, STAT1			FOS	STAT2, IRF3	JUN, RELA	JUN, STAT5A		FOS, RELA	JUN, RELA	FOS, TP53	

Table summarizing measles infection pathway-related transcriptional regulators recovered by ISMARA, Lisa and *xcore* among their top-scoring signatures based on rinderpest infection datasets. The list of measles infection pathway-related transcriptional regulators used to assess the recovery was constructed using KEGG pathway *Measles* (map05162) by including only regulators available in the references of all tools

**Table 3:** Recovering heart development transcriptional regulators

Top signatures	ISMARA	Lisa	*xcore*	
			ReMap2020	ChIP-Atlas
1–10		GATA6, SMAD3, GATA4		
11–50	SNAI2, MEF2A, SRF, GATA4	SMAD1, EOMES, GATA3, SMAD2	SMAD3, NKX2-5, ATF2, TBX5, RBPJ	RARA
51–100	MEF2C, WT1	TBX5, REST, MBD2, TP53, SMAD4	SNAI2	JUN, TP53

Table summarizing heart development-related transcriptional regulators recovered by ISMARA, Lisa and *xcore* among their top-scoring signatures based on Human H3 embryonic stem cells differentiated to cardiomyocytes time-series dataset. The list of heart development-related transcriptional regulators used to assess the recovery was constructed using Gene Ontology term *heart development* (GO:0007507) by including only regulators available in the references of all tools

## Conclusions

*Xcore* provides a flexible framework for integrative analysis of gene expression and publicly available TF binding data to unravel putative transcriptional regulators and their activities. Our analyses showed superior results when using ChIP-seq based signatures as compared to motifs-based ones. We attribute this difference to the presence of biotype-specific binding information which might be lost in motifs that describe more general transcription factor binding preferences. Despite high numbers of ChIP-seq signatures and redundancy, our machine learning framework is able to select biologically relevant signatures. In our comparison with motif-based ISMARA and ChIP-seq based Lisa, *xcore* performed competitively with those tools. Especially, both *xcore* and Lisa worked exceptionally well at recovering EMT-related transcriptional regulators. However, a comprehensive comparison of *xcore* with other tools would require further benchmarking efforts. Such efforts are currently hindered by the lack of standard bench-marking datasets for transcriptional regulators’ inference problems. In conclusion, *xcore* is useful for generating testable hypotheses about the data and provides a novel way to connect gene expression data with relevant ChIP-seq experiments.

## Methods

### TGF-β1 stimulation to A-549/MDA-231-D

A-549 Lung cancer cells (CCL-185, ATCC) and MDA-231-D highly metastatic human breast cancer cells [26] (gift from Dr. Kohei Miyazono, Tokyo Univ.) were cultured in Dulbecco’s modified Eagle’s medium (Thermo Fisher Scientific Inc., Waltham, MA, USA) supplemented with 10% fetal bovine serum, 1 mM sodium pyruvate (Thermo Fisher Scientific Inc., Waltham, MA, USA) and penicillin/streptomycin (100 U/mL, 100 µg/mL; Thermo Fisher Scientific Inc., Waltham, MA, USA). TGF-β1 (7754-BH, Recombinant Human TGF-beta 1, R&D Systems) was added at the final concentration of 1 ng/mL. At 0, 1, 2, 4, 6, and 24 h post stimulation, cells were harvested followed by RNA extraction using RNeasy mini kit (Qiagen, Valencia, CA, USA). Transcriptome data was produced by nAnT-iCAGE [27]. CAGE libraries were sequenced on Illumina HiSeq 2500 (50-nt single read).

## Supplementary Information

**Additional file 1: Figure S1**. (A) Boxplots showing R^2^ values for gene expression prediction models constructed either on gene- or promoter-level expression data. Each boxplot shows R^2^ values pooled across all the replicates. Models were trained and evaluated in tenfold cross-validation on individual replicates, using data on gene expression changes between 0 and 24 h after treatment in our newly generated TGFβ induced EMT experiment performed in A-549 and MDA-231-D cell lines. The models were constructed using ReMap2020 or ChIP-Atlas molecular signatures.

**Additional file 2: Figure S2**. (A) Heatmap showing the dynamics of TF activities during TGFβ induced EMT. Heatmaps on the left present TF activities estimated using CAGE data from our newly generated TGFβ induced EMT experiment performed on A-549 and MDA-231-D cell lines. Heatmap on the right depicts TF activities estimated using previously published microarray data from the TGFβ induced EMT experiment performed on A-549 cell lines. The TF activities were calculated in the reference to 0 h time point. Only the top-scoring ChIP-Atlas signatures are shown. Grey color designates NA values. (B) Boxplots showing R^2^ values for gene expression prediction models constructed using different molecular signature sets: Motif based (Jaspar, SwissRegulon) and ChIP-seq based (ReMap2020, ChIP-Atlas). Each boxplot shows R^2^ values pooled across all the replicates. Models were trained and evaluated in tenfold cross-validation on individual replicates, using data on gene expression changes between 0 and 24 h after the rinderpest infection treatment experiment performed in 293SLAM cell line. (C) Boxplots showing R^2^ values for gene expression prediction models trained using lasso, elastic net or ridge regression method. Each boxplot shows R^2^ values pooled across all the replicates. Models were trained and evaluated in tenfold cross-validation on individual replicates, using data on gene expression changes between 0 and 24 h after treatment in our newly generated TGFβ induced EMT experiment performed in A-549 and MDA-231-D cell lines. The models were constructed using ReMap2020 molecular signatures and promoter-level expression data.

**Additional file 3:** **Extended Materials and Methods.** Extended description of procedures used to process the raw CAGE data, construct molecular signatures, and assess the accuracy of used models.

**Additional file 4: Table S1**. Table provides the activities of ReMap2020 and ChIP-Atlas molecular signatures estimated using TGFβ induced EMT in A-549 cell line dataset.

**Additional file 5: Table S2**. Table provides the activities of ReMap2020 and ChIP-Atlas molecular signatures estimated using TGFβ induced EMT in MDA-231-D cell line dataset.

**Additional file 6: Table S3**. Table provides the activities of ReMap2020 and ChIP-Atlas molecular signatures estimated using TGFβ induced EMT in A-549 cell line dataset (GSE17708).

**Additional file 7: Table S4**. Table provides the activities of ReMap2020 and ChIP-Atlas molecular signatures estimated using rinderpest infection in 293SLAM and COBL-a cell lines datasets.

**Additional file 8: Table S5**. Table provides the activities of ReMap2020 and ChIP-Atlas molecular signatures estimated using Human H3 embryonic stem cells differentiated to cardiomyocytes time-course.

## Data Availability

Xcore and xcoredata R packages are open-source and freely available on GitHub under https://github.com/bkaczkowski/xcore and https://github.com/mcjmigdal/xcoredata. xcore user guide is available https://bkaczkowski.github.io/xcore/articles/xcore_vignette.html. Official releases of xcore and xcoredata packages are also included in the Bioconductor, from where they can be easily installed using BiocManager functionality. The EMT datasets generated and/or analyzed during the current study are available in the NCBI SRA repository: https://www.ncbi.nlm.nih.gov/geo/query/acc.cgi?acc=GSE17708 and https://www.ncbi.nlm.nih.gov/bioproject/879326. The rinderpest infection dataset analyzed during the current study is available in the FANTOM5 catalog: https://fantom.gsc.riken.jp/5/sstar/Rinderpest_infection_series. The Human H3 embryonic stem cells differentiated to cardiomyocytes dataset analyzed during the current study is available in the FANTOM5 catalog: https://fantom.gsc.riken.jp/5/sstar/ES_to_cardiomyocyte.

## Abbreviations

CAGE: Cap analysis gene expression
CPM: Counts per million
CV: Cross-validation
EMT: Epithelial-mesenchymal transition
TF: Transcription factor
TFBS: Transcription factor binding site
TGFβ: Transforming growth factor beta

## Availability and requirements

Project name: xcore. Project home page: https://github.com/bkaczkowski/xcore. Archived version: 1.1.4. Operating system: Platform independent. Programming language: R. Other requirements: None. License: GPL-2. Any restrictions to use by non-academics: No restrictions.
