# Supplementary figures and images for: xcore: an R package for inference of gene expression regulators
Source: BMC Bioinformatics. 2023 Jan 11;24:14. doi: 10.1186/s12859-022-05084-0 (PMC9832628; doi:10.1186/s12859-022-05084-0)

A

A-549

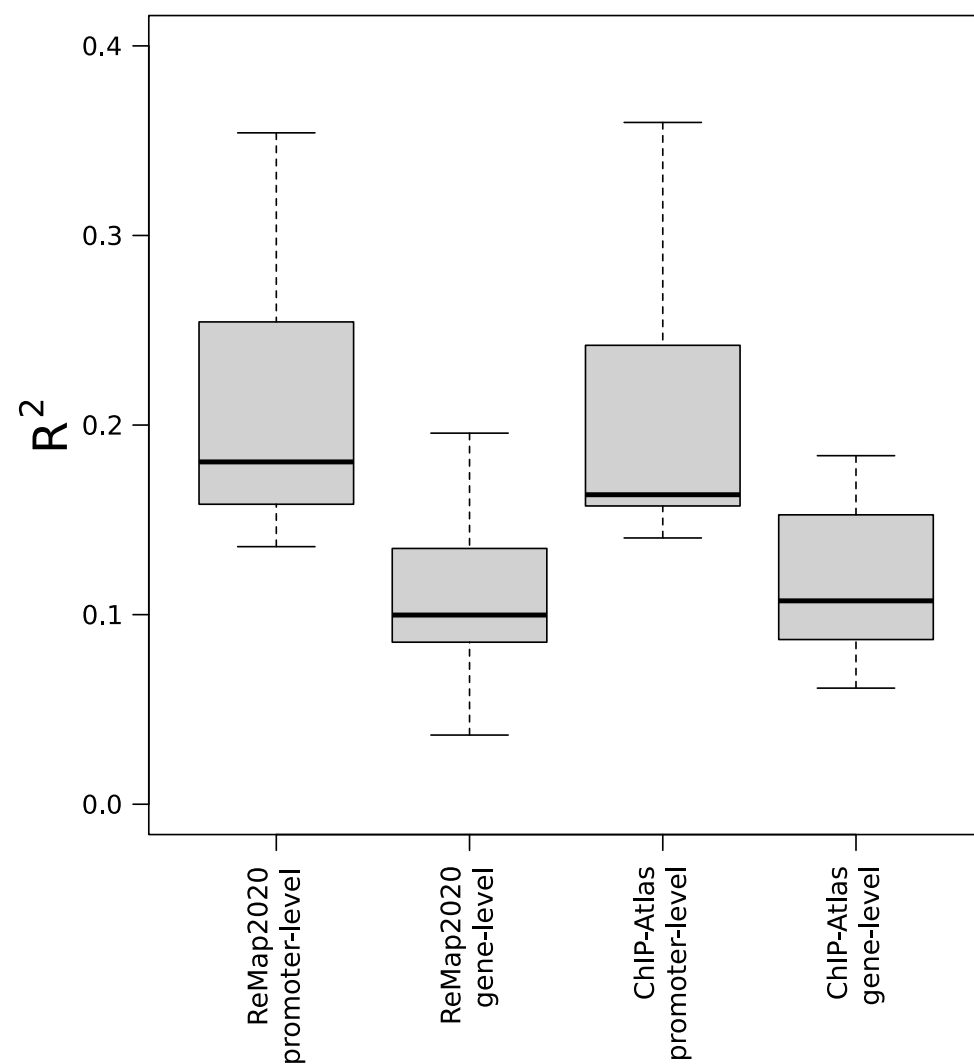

MDA-321-D

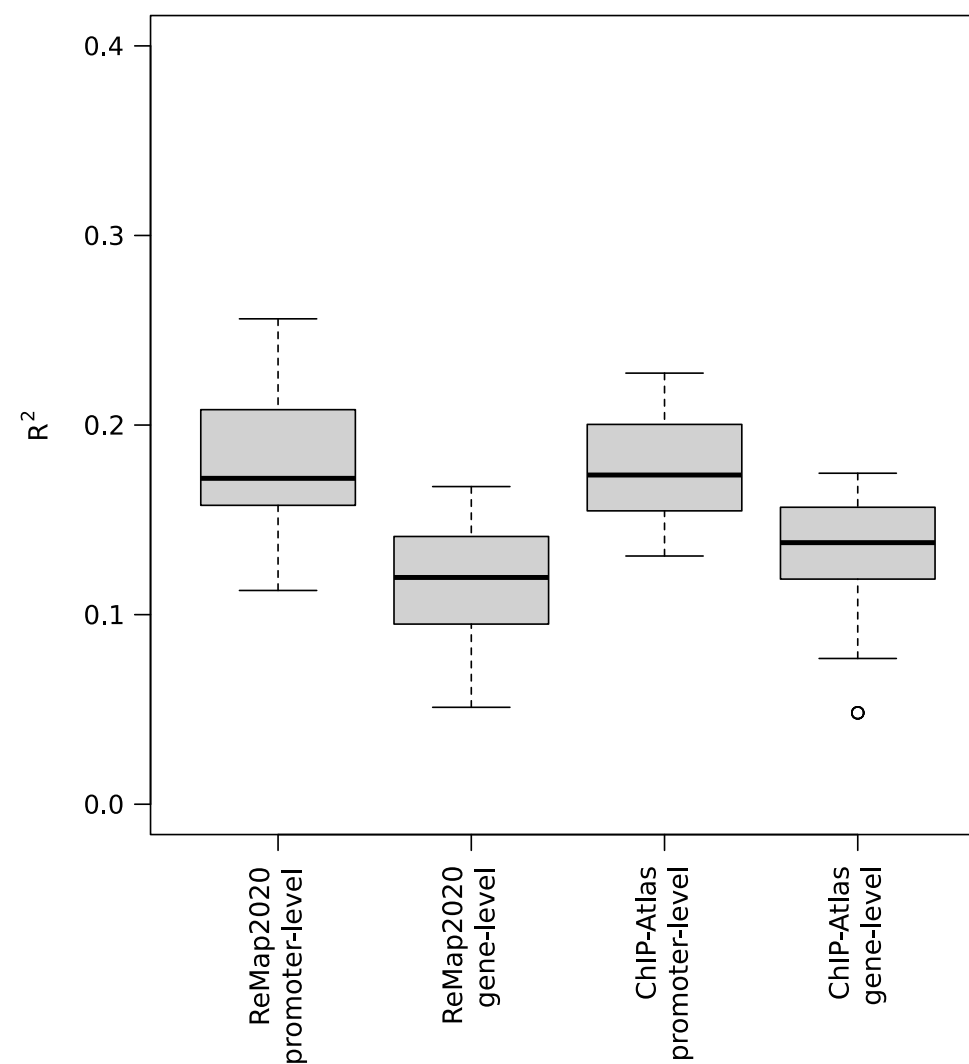

Supplement: Supplementary file 1 — Additional file 1: Figure S1. (A) Boxplots showing R2 values for gene expression prediction models constructed either on gene- or promoter-level expression data. Each boxplot shows R2 values pooled across all the replicates. Models were trained and evaluated in tenfold cross-validation on individual replicates, using data on gene expression changes between 0 and 24 h after treatment in our newly generated TGFβ induced EMT experiment performed in A-549 and MDA-231-D cell lines. The models were constructed using ReMap2020 or ChIP-Atlas molecular signatures. [file 12859_2022_5084_MOESM1_ESM.pdf]

**A**

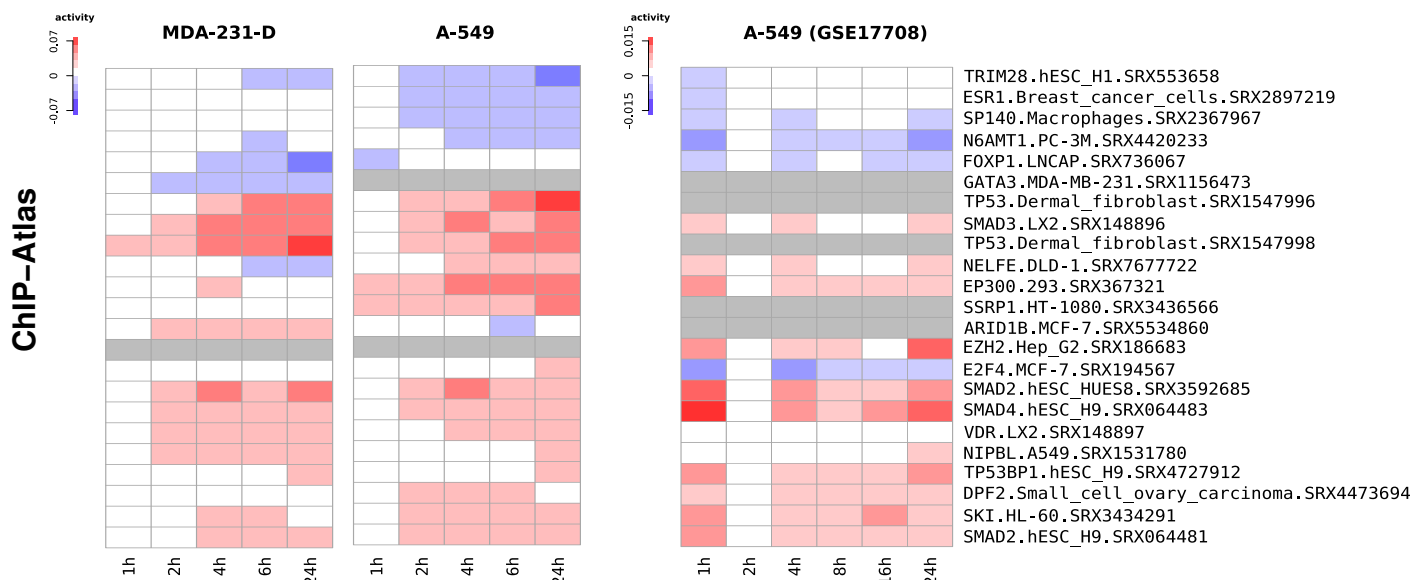

**B**

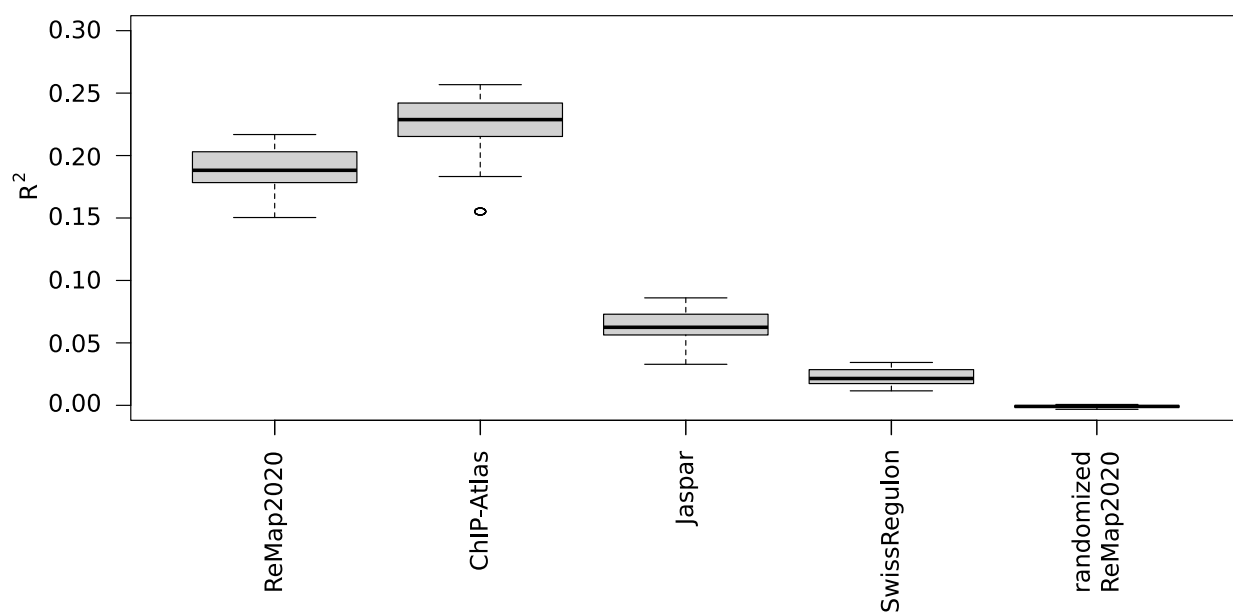

**C**

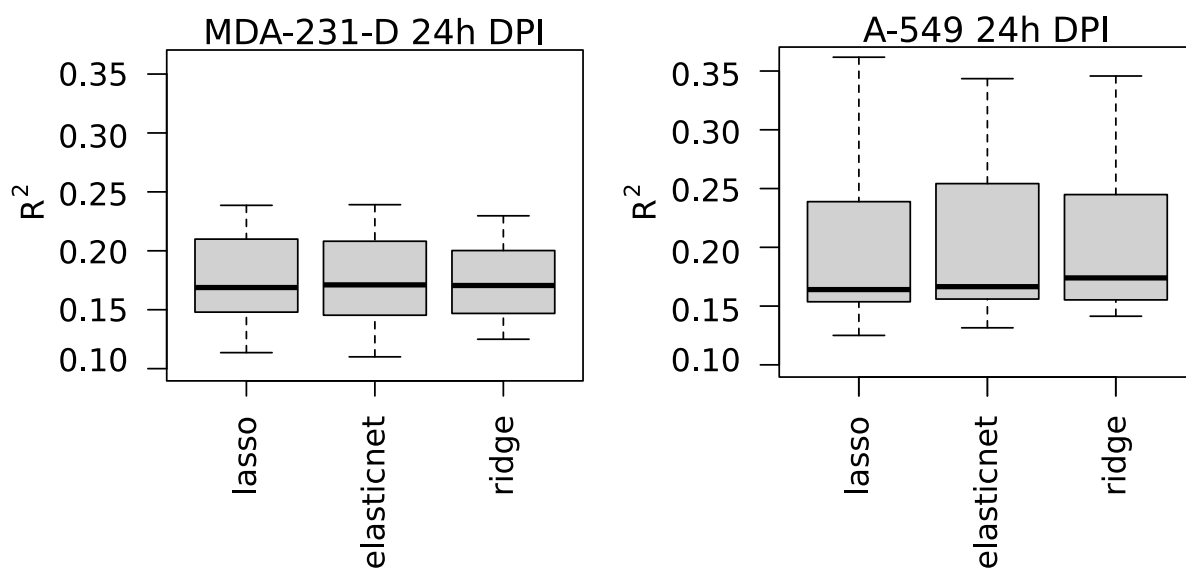

Supplement: Supplementary file 2 — Additional file 2: Figure S2. (A) Heatmap showing the dynamics of TF activities during TGFβ induced EMT. Heatmaps on the left present TF activities estimated using CAGE data from our newly generated TGFβ induced EMT experiment performed on A-549 and MDA-231-D cell lines. Heatmap on the right depicts TF activities estimated using previously published microarray data from the TGFβ induced EMT experiment performed on A-549 cell lines. The TF activities were calculated in the reference to 0 h time point. Only the top-scoring ChIP-Atlas signatures are shown. Grey color designates NA values. (B) Boxplots showing R2 values for gene expression prediction models constructed using different molecular signature sets: Motif based (Jaspar, SwissRegulon) and ChIP-seq based (ReMap2020, ChIP-Atlas). Each boxplot shows R2 values pooled across all the replicates. Models were trained and evaluated in tenfold cross-validation on individual replicates, using data on gene expression changes between 0 and 24 h after the rinderpest infection treatment experiment performed in 293SLAM cell line. (C) Boxplots showing R2 values for gene expression prediction models trained using lasso, elastic net or ridge regression method. Each boxplot shows R2 values pooled across all the replicates. Models were trained and evaluated in tenfold cross-validation on individual replicates, using data on gene expression changes between 0 and 24 h after treatment in our newly generated TGFβ induced EMT experiment performed in A-549 and MDA-231-D cell lines. The models were constructed using ReMap2020 molecular signatures and promoter-level expression data. [file 12859_2022_5084_MOESM2_ESM.pdf]
