# Supplementary material for: xcore: an R package for inference of gene expression regulators
Source: BMC Bioinformatics. 2023 Jan 11;24:14. doi: 10.1186/s12859-022-05084-0 (PMC9832628; doi:10.1186/s12859-022-05084-0)
Supplement: Supplementary file 3 — Additional file 3: Extended Materials and Methods. Extended description of procedures used to process the raw CAGE data, construct molecular signatures, and assess the accuracy of used models. [file 12859_2022_5084_MOESM3_ESM.docx]

**Extended Materials and Methods**

**Expression data processing**

Raw sequencing CAGE data were processed using MOIRAI pipeline [1]. In short, TagDust2 was used to trim the adapter sequences and trimmed reads were then mapped to the human genome using BWA aligner. Uniquely mapping reads with overlapping 5’ ends overlapping the coordinates of the FANTOM5 robust promoter set [2] were counted (raw expression counts). Counts for rinderpest infection time-course were obtained from FANTOM5’s atlas [3]. For the microarray dataset we downloaded the log-transformed normalized data (GSE17708) [4].

For CAGE datasets we consider two levels of expression data, promoter level where the expression is measured at all FANTOM5’ promoter regions [3] and gene level (HUGO gene symbol) where for each gene we use expression at its highest scored FANTOM5’ promoter region with an GENCODE 38 annotation [5] and ROADMAP promoter confirmation [6]. Expression tags for each sample were filtered to exclude lowly expressed promoters, normalized for the library size and transformed into counts per million (CPM) using edgeR [7]. Next, CPM were log2 transformed with addition of pseudo count 1. For the microarray dataset we consider only the gene level expression data using already pre-normalized log-transformed data. Individual probes were matched to FANTOM5’ promoter regions based on their ENTREZID. For each dataset we designate the base level samples, taken as an earliest point in the time series, for which we calculate per gene mean expression over all replicates, further called basal expression level.

**Molecular signatures generation**

We downloaded sets of peaks for all human transcription factors for human genome assembly hg38 from ReMap2020 [8] and ChIP-Atlas [9] databases. For ReMap2020 no further processing of peaks was applied. In the case of ChIP-Atlas we excluded ambiguous experiments such as those labeled as "Epitope tags" or “Biotin”. The molecular signatures were constructed by first extending FANTOM5’ promoter regions by 500 bp in both directions. Next, peaks were intersected with promoter regions yielding a molecular signature, where 1 indicates presence of a signature in the promoter and 0 indicates its absence.

Additionally, we considered molecular signatures based on predicted transcription factors binding sites from Jaspar [10] and SwissRegulon [11] databases.

**Assessing Model Accuracy**

To assess models accuracy we calculate R^2^ using the following formula:

$$R^{2}=1-\frac{RSS}{TSS}=1-\frac{\sum_{i}^{n} (y_{i}-\hat{y}_{i})^{2}}{\sum_{i}^{n} (y_{i}-\bar{y})^{2}}$$

where, RSS - residual sum of squares, TSS - total sum of squares, y_i_ - i-th promoter expression, $\hat{y}$- i-th promoter predicated expression, $\bar{y}$ - mean promoter expression. For each model R^2^ is calculated using 10-fold cross-validation separately for each biological replicate, finally estimates are averaged across replicates.

**References**

1. Hasegawa A, Daub C, Carninci P, Hayashizaki Y, Lassmann T. MOIRAI: a compact workflow system for CAGE analysis. BMC Bioinformatics. 2014;15:144.

2. Forrest ARR, Kawaji H, Rehli M, Kenneth Baillie J, de Hoon MJL, Haberle V, et al. A promoter-level mammalian expression atlas. Nature. 2014;507:462–70.

3. Lizio M, Harshbarger J, Shimoji H, Severin J, Kasukawa T, Sahin S, et al. Gateways to the FANTOM5 promoter level mammalian expression atlas. Genome Biol. 2015;16:22.

4. Sartor MA, Mahavisno V, Keshamouni VG, Cavalcoli J, Wright Z, Karnovsky A, et al. ConceptGen: a gene set enrichment and gene set relation mapping tool. Bioinformatics. 2010;26:456–63.

5. Frankish A, Diekhans M, Jungreis I, Lagarde J, Loveland JE, Mudge JM, et al. GENCODE 2021. Nucleic Acids Res. 2021;49:D916–23.

6. Kundaje A, Meuleman W, Ernst J, Bilenky M, Yen A, Heravi-Moussavi A, et al. Integrative analysis of 111 reference human epigenomes. Nature. 2015;518:317–30.

7. Robinson MD, McCarthy DJ, Smyth GK. edgeR: a Bioconductor package for differential expression analysis of digital gene expression data. Bioinformatics. 2010;26:139–40.

8. Chèneby J, Ménétrier Z, Mestdagh M, Rosnet T, Douida A, Rhalloussi W, et al. ReMap 2020: a database of regulatory regions from an integrative analysis of Human and Arabidopsis DNA-binding sequencing experiments. Nucleic Acids Res. 2020;48:D180–8.

9. Oki S, Ohta T, Shioi G, Hatanaka H, Ogasawara O, Okuda Y, et al. ChIP-Atlas: a data-mining suite powered by full integration of public ChIP-seq data. EMBO Rep. 2018;19:e46255.

10. Fornes O, Castro-Mondragon JA, Khan A, van der Lee R, Zhang X, Richmond PA, et al. JASPAR 2020: update of the open-access database of transcription factor binding profiles. Nucleic Acids Res. 2020;48:D87–92.

11. Pachkov M, Balwierz PJ, Arnold P, Ozonov E, van Nimwegen E. SwissRegulon, a database of genome-wide annotations of regulatory sites: recent updates. Nucleic Acids Res. 2013;41:D214–20.
